# Immunohistochemical Characterization of Spermatogenesis in the Ascidian *Ciona robusta*

**DOI:** 10.3390/cells13221863

**Published:** 2024-11-11

**Authors:** Haruka Sakurai, Kogiku Shiba, Katsumi Takamura, Kazuo Inaba

**Affiliations:** 1 Shimoda Marine Research Center, University of Tsukuba, 5-10-1, Shimoda 415-0025, Shizuoka, Japan; 2 Faculty of Life Science and Biotechnology, Fukuyama University, Fukuyama 729-0292, Hiroshima, Japan

**Keywords:** ascidian sperm, spermatogenesis, cilia and flagella, Sertoli cell

## Abstract

Animals show diverse processes of gametogenesis in the evolutionary pathway. Here, we characterized the spermatogenic cells in the testis of the marine invertebrate *Ciona robusta. Ciona* sperm differentiate in a non-cystic type of testis, comprising many follicles with various sizes and stages of spermatogenic cells. In the space among follicles, we observed free cells that were recognized by antibody against Müllerian inhibiting substance, a marker for vertebrate Sertoli cells. We further categorized the spermatogenic cells into four round stages (RI to RIV) and three elongated stages (EI to EIII) by morphological and immunohistochemical criteria. An antibody against a vertebrate Vasa homolog recognized a few large spermatogonium-like cells (RI) near the basal wall of a follicle. Consistent with the period of meiosis, a synaptonemal complex protein SYCP3 was recognized from early spermatocytes (RII) to early spermatids (E1). Acetylated tubulins were detected in spermatids before flagellar elongation at the RIV stage and became distributed along the flagella. Electron microscopy showed that the free cells outside the testicular follicle possessed a characteristic of vertebrate Sertoli cells. These results would provide a basis for basic and comparative studies on the mechanism of spermatogenesis.

## 1. Introduction

Spermatogenesis is a highly organized process that generates haploid sperm cells through the proliferation, meiosis, and differentiation of germ cells, accompanied by a significant morphological transformation known as spermiogenesis. Diploid spermatogonia function as stem cells, continuously replenishing the spermatogonial pool. A subset of these cells differentiates into subsequent stages of spermatogenic cells, including spermatocytes, spermatids, and, ultimately, mature sperm. In addition to these germline cells, two types of somatic cells, Sertoli and Leydig cells, play crucial roles in regulating the development and differentiation of the germ cell lineage [1,2].

In mammals, spermatogenesis takes place in the seminiferous tubules, where it progresses from the basal lamina toward the lumen. In anamniote vertebrates, such as fish and frogs, spermatogenesis occurs within cysts, each containing a specific stage of developing spermatogenic cell syncytia. Each cyst is enclosed by a layer of Sertoli cells [3]. Ascidians, marine invertebrates, provide valuable insights into vertebrate evolution [4] and present unique models for understanding gamete function [5,6]. For instance, *Ciona* sperm exhibit clear activation of motility and chemotaxis toward the egg during fertilization [7,8,9]. The molecular mechanisms underlying intracellular Ca^2+^ dynamics and dynein regulation during fertilization have been elucidated in *Ciona* sperm [10,11]. In ascidian sperm, the mitochondrion is positioned laterally on the nucleus, and during a distinctive event known as sperm reaction, the mitochondrion translocates along the flagellum, contributing to the exclusion of paternal mitochondria from embryonic development [12,13]. Moreover, ascidians are hermaphroditic and exhibit gamete self-incompatibility mechanisms to prevent self-fertilization [14,15,16]. Thus, *Ciona* sperm serve as a unique system for studying sperm behavior and sperm-egg interactions during fertilization.

Previous studies have reported that gonad rudiment formation begins early in juvenile stages after metamorphosis and subsequently differentiates into the ovotestis [17,18,19,20]. The later stages of spermatogenesis have been described through light and electron microscopy [17,21,22,23,24]. However, despite significant advancements in understanding sperm physiology, the process of sperm formation in *Ciona* remains less well understood, and somatic cells supporting spermatogenesis, such as Sertoli and Leydig cells, have yet to be identified. We previously identified a set of genes highly and uniquely expressed in the testis, termed testis-expressed class D (TD) genes [5,25]. In this paper, we characterize spermatogenic cells in *Ciona robusta* using immunohistochemical methods, employing antibodies against two TD gene products.

## 2. Materials and Methods

### 2.1. Dissection of Testis Tissue from Adult Animals

Adults of *Ciona robusta* were cultivated either at the National Bioresource Project (NBRP) within the Field Science Education and Research Center, Maizuru Fisheries Experimental Station, Kyoto University (Maizuru City, Kyoto Prefecture), or at the Misaki Marine Biological Station, The University of Tokyo (Miura City, Kanagawa Prefecture). In some experiments, wild adults were collected at the Tohoku University Graduate School of Agricultural Science’s Complex Ecology Field Education and Research Center (Onagawa Town, Miyagi Prefecture) and transferred to an aquarium at the Shimoda Marine Research Center, University of Tsukuba. To prevent spawning, the specimens were maintained under controlled lighting conditions at seawater temperatures below 18 °C. Testes were dissected using dissection scissors and removed from the gut wall with ophthalmic forceps. The collected testes were preserved in filtered seawater on ice.

### 2.2. Isolation of Spermatogenic Cells

Testes from 3 to 5 individuals were placed in a 1.5 mL tube containing three volumes of filtered seawater. The tissue was gently minced with ophthalmic forceps, and the mixture was tapped to suspend the contents. The sample was briefly centrifuged at 1260× *g* for 10 s to separate the tissue fragments. Spermatogenic cells were collected from the supernatant. All procedures were performed on ice.

### 2.3. Histological Observation of Testes

The dissected testes were fixed overnight in Bouin’s solution, and then transferred to 70% ethanol. The tissues were dehydrated through an ethanol series, replaced with xylene, and embedded in paraffin. Sections of 8 μm thickness were prepared and stained with Hematoxylin and Eosin (HE).

### 2.4. Preparation of Frozen Sections

Frozen sections were prepared from both unfixed and PFA-fixed testes. Dissected testes were washed in artificial seawater (ASW) and then placed in embedding medium White Tissue Coat (WTC, Yuai Kasei Co., Amagasaki, Japan) at 4 °C for 1 h before being frozen in MC802. For PFA fixation, testes were washed in ASW, then in 0.1 M MOPS and 0.5 M NaCl, and fixed in 4% PFA in phosphate-buffered saline (PBS) at 4 °C overnight. The fixed testes were washed three times for 20 min each in PBS, and then sequentially incubated in 10% sucrose in PBS at 4 °C for 30 min, 15% sucrose in PBS at 4 °C for 30 min, and 20% sucrose in PBS at 4 °C overnight. After an additional 30 min in 20% sucrose in PBS, the tissues were embedded in WTC at 4 °C for 1 h and frozen in MC802. Sections of 4 μm thickness were cut using a cryostat (REM-710 Retratome equipped with MC802, Yamato Kohki, Asaka, Japan), mounted on MAS-coated slides (Matsunami Glass Ind., Kishiwada, Japan), air-dried at room temperature for 1 h, and used for immunofluorescence staining. Samples not used immediately were stored at −80 °C.

### 2.5. Primary Antibodies

For immunofluorescence analysis, we used five primary antibodies at the following dilutions: Anti-CiVH antibody (mouse monoclonal, 1:100) [20]; Anti-TD09 antibody (mouse polyclonal, 1:100) [25]; Anti-TD02 antibody (mouse polyclonal, 1:100) [25]; Anti-acetylated α-tubulin antibody (T6793, Sigma-Aldrich Co., Japan, mouse monoclonal, 1:1000); and Anti-MIS antibody H-300 (SC-28912, Biotechnology Inc., Santa Cruz, TX, USA, rabbit polyclonal, 1:1000).

### 2.6. Immunohistochemistry

Spermatogenic cells adhered to MAS-coated slides were washed with a buffer containing 0.1 M MOPS and 0.5 M NaCl, and fixed with 4% paraformaldehyde in PBS at room temperature for 1 h, followed by fixation with 100% methanol at −20 °C for 10 min. The samples were quickly dried with cold air and rehydrated in PBS at room temperature for 5 min or more. Frozen sections, either freshly prepared or stored at −80 °C, were air-dried at room temperature for 1 h, fixed with 100% methanol at −20 °C for 10 min, quickly dried with cold air, and rehydrated in PBS for 5 min or more. The sections were washed three times in PBS to remove WTC. Blocking was performed with 10% goat serum in PBS, followed by incubation with primary antibodies at room temperature for 1 h or at 4 °C overnight. After washing with 0.05% Triton X-100 in PBS (T-PBS) or PBS three times for 5 min each, sections were incubated with secondary antibodies (goat Alexa-546-conjugated anti-mouse or anti-rabbit antibody, Thermo Fisher Scientific Inc., Tokyo, Japan). Nuclear staining was performed with 10 μM DAPI in PBS. Images were captured and analyzed using a fluorescent microscope with differential interference contrast objectives (BX51, Olympus Corp., Tokyo, Japan). The stages of spermatogenic cells were determined according to the criteria shown in Table 1.

### 2.7. Electron Microscopy

Trimmed testis tissues were fixed in 0.45 M sucrose, 2.5% glutaraldehyde, and 0.1 M sodium cacodylate (pH 7.2) at 4 °C for 2 h, postfixed with 1% OsO_4_ buffered in 0.1 M sodium cacodylate (pH 7.2) at 4 °C for 2 h, and dehydrated through a graded ethanol and propylene oxide series. Samples were embedded in Quetol 812 (Nisshin EM Co., Tokyo, Japan), sectioned, stained with uranyl acetate and lead citrate, and observed under a transmission electron microscope (JEM 1010EX; JEOL, Tokyo, Japan), as described previously [26].

## 3. Results

### 3.1. Observation and Classification of Spermatogenic Cells in Ciona Testis

**Figure 1:**
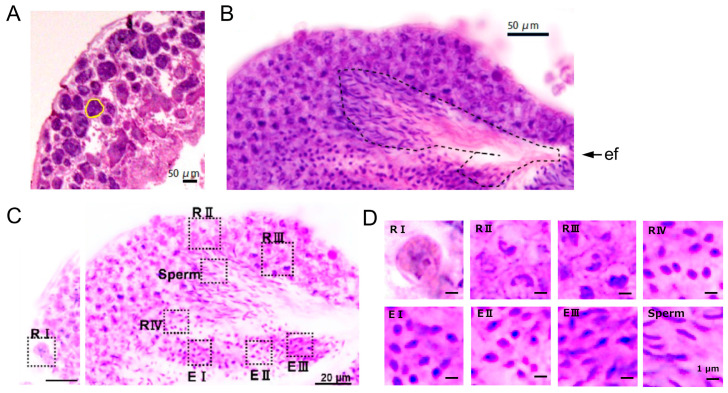
Paraffin section images of testicular follicles in the ascidian *Ciona robusta.* (**A**) A section of the testis, showing that it is composed of numerous testicular follicles (yellow circle). (**B**) Distribution of spermatogenic cells within a testicular follicle. Cells with round nuclei are located in the peripheral region, while mature sperm are seen in the central region, connected to the efferent duct (dashed line). Abbreviation: ef, efferent duct. (**C**,**D**) Localization of different stages of spermatogenic cells within a follicle. The areas highlighted by dashed squares in (**C**) are shown at higher magnification in (**D**).

We initially examined HE-stained paraffin sections of the testes. The *Ciona* testis consists of numerous club-like compartments known as testicular follicles (Figure 1A). Each follicle was densely packed with spermatogenic cells at various developmental stages. As previously observed in *Ciona* [21] and *Botryllus* [23], spermatogenic cells were distributed from larger cells near the basal lamina to smaller, later-stage cells in the center, indicating that the testis is non-cystic rather than cystic. However, the alignment of spermatogenic cells is less strictly organized compared to the mammalian seminiferous tubules. Late spermatids and mature sperm were observed in the region from the center to the efferent duct (Figure 1B). Based on cell size (Figure 1C,D) and the immunohistochemical characterizations described below, we classified the spermatogenic cells into round stages (RI to RIV) and elongated stages (EI to EIII) (Table 1).

### 3.2. Classification of Spermatogenic Cells Using Immunohistochemical Staining

#### 3.2.1. MIS H-300 Antibody

**Figure 2:**
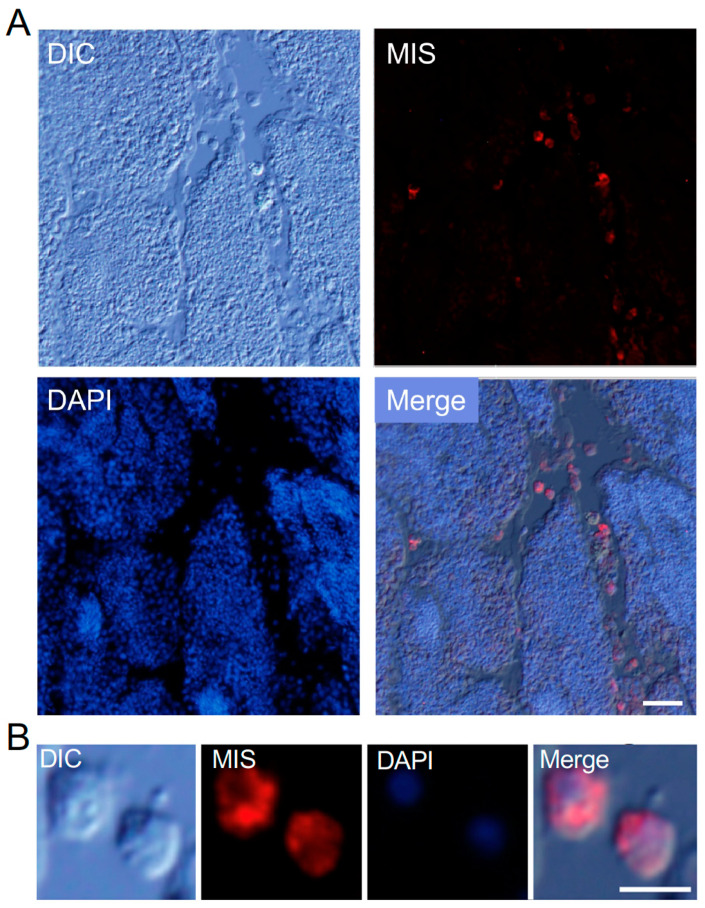
Immunofluorescence image of a frozen section stained with anti-Müllerian Inhibiting Substance (MIS) antibody. (**A**) MIS H-300 positive cells (red) are localized in the space between testicular follicles. Scale bar, 20 μm. (**B**) The cells located between follicles display a round shape. Scale bar, 5 μm. Blue, DAPI staining.

Sertoli cells are essential for the growth and differentiation of spermatogenic cells in mammalian testes. However, previous studies on *Boltenia villosa* suggested the absence of Sertoli-like cells in ascidians [22]. To investigate the presence of Sertoli-like cells and potential interactions between germ cells and somatic cells in *Ciona*, we used the MIS H-300 antibody, which targets Müllerian Inhibiting Substance (MIS), a TGFβ superfamily member secreted by Sertoli cells in vertebrates. Immunofluorescence staining revealed that free cells located between testicular follicles were positively stained with the MIS H-300 antibody (Figure 2A), while no spermatogenic cells within the follicles were stained. Further analysis of isolated cells showed that the signal was localized in the cytoplasm of these free cells, particularly near the nucleus (Figure 2B).

#### 3.2.2. Anti-CiVH Antibody

**Figure 3:**
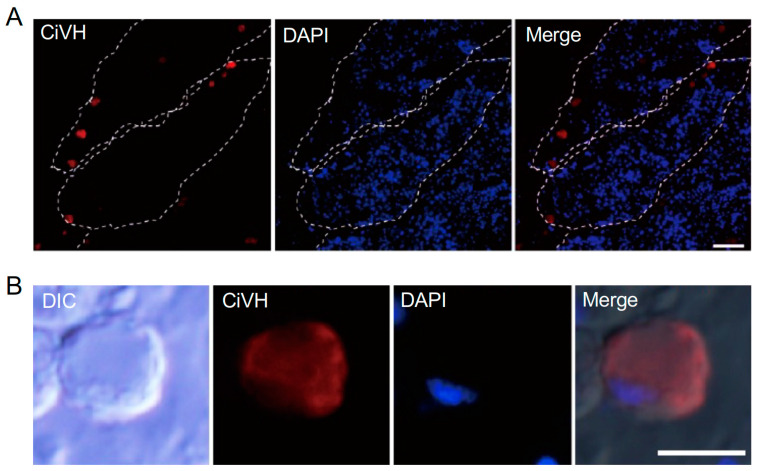
Immunofluorescence image of a frozen section stained with an antibody against *Ciona robusta* Vasa homolog (CiVH). (**A**) CiVH signals (red) are detected in a few large cells located along the basal region of a testicular follicle. Scale bar, 20 μm. (**B**) CiVH-positive cells appear round and large, likely representing primordial germ cells or spermatogonia (RI). The immunofluorescence signal was present throughout the cytoplasm but not in the nucleus (blue). Scale bar, 5 μm.

Vasa protein is a marker for primordial germ cells and spermatogonia. The anti-CiVH antibody, a monoclonal antibody that recognizes primordial germ cells and early-stage germ cells in *Ciona* juveniles [20], was used to stain adult *Ciona* testes. Immunofluorescence revealed a few large cells along the walls of the testicular follicles (Figure 3A). These isolated cells appeared round, with a diameter greater than 7.3 μm, and the immunofluorescence signal was present throughout the cytoplasm but not in the nucleus (Figure 3B).

#### 3.2.3. Anti-TD09 Antibody

**Figure 4:**
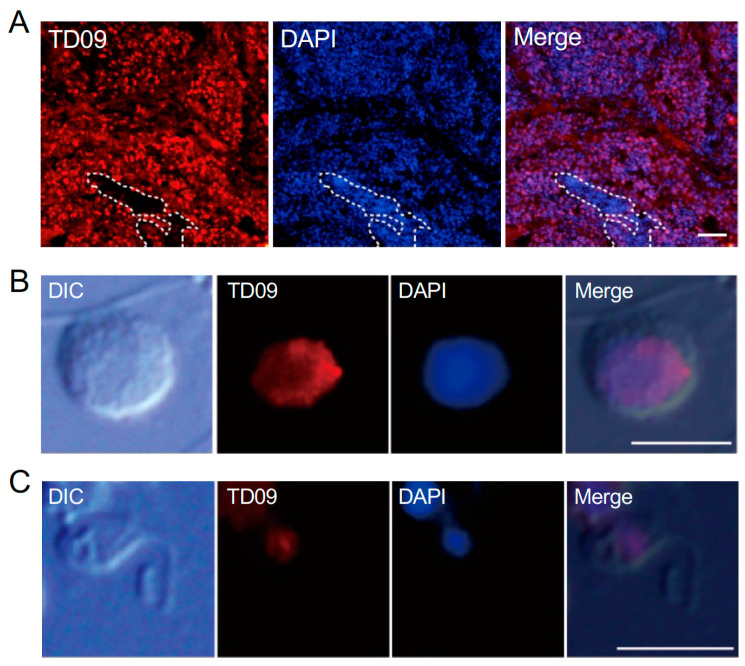
Immunofluorescence image of a frozen section stained with an antibody against TD09. This protein is one of the gene products from highly expressed genes in *Ciona* testis, showing homology with the synaptonemal complex protein SYCP3. (**A**) The antibody recognizes a broad area of the testis (red), except for the central region near the efferent duct containing mature sperm. Scale bar, 20 μm. (**B**,**C**) The anti-TD09 antibody detects the nuclei of both round-stage cells (RII to RIV; (**B**)) and short-flagellated elongated-stage cells (EI; (**C**)). Scale bar, 5 μm. Blue, DAPI staining.

The TD09 gene, highly expressed in Ciona testis, is homologous to the synap-tonemal complex protein SYCP3, which is expressed in meiotic cells [5,25,27]. An an-tibody developed against TD09 recognized broad areas of the testis section, excluding the region containing mature sperm near the efferent duct (Figure 4A). In isolated spermatogenic cells, the nuclei of round-stage cells (RII to RIV) and short-flagellated elongated cells (EI) were clearly stained (Figure 4B,C). TD09-positive cells in the round stages ranged in diameter from 3.2 μm to 6.2 μm, while the flagella of elongated cells were 5.0 μm or less.

#### 3.2.4. Anti-TD02 Antibody

**Figure 5:**
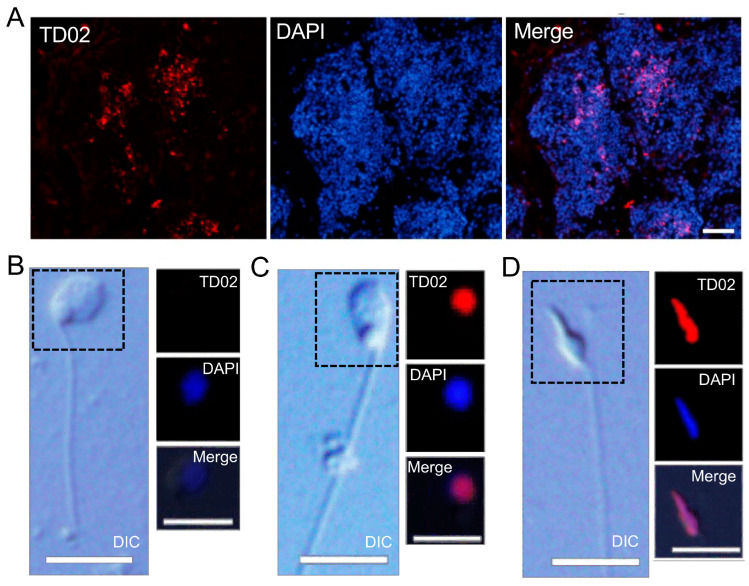
Immunofluorescence image of a frozen section stained with an antibody against TD02. This protein is one of the gene products from highly expressed genes in *Ciona* testis, showing homology with histone H1-like protein or protamine. (**A**) The antibody recognizes spermatids and mature sperm (red) located in the central area of the testicular follicle near the efferent duct. Scale bar, 20 μm. (**B**–**D**) The antibody stained the nuclei of late spermatids and mature sperm (EII; (**C**), EIII; (**D**)) but not early-stage spermatids (EI; (**B**)), even though the latter had already formed long flagella. Scale bar, 5 μm. Red, TD02 immunostaining. Blue, DAPI staining.

TD02 is another gene highly expressed in *Ciona* testis, showing homology to a histone H1-like protein involved in chromatin condensation during spermiogenesis [5,25]. An antibody against TD02 stained the nucleus of late spermatids and mature sperm in the central area of the testicular follicle near the efferent duct (Figure 5A). In isolated cells, the antibody recognized the nucleus of late spermatids and mature sperm but did not stain early spermatids (E1), even those with long flagella (Figure 5B–D). The appearance of the TD02 protein coincided with the onset of nuclear elongation, which began in spermatids with flagella measuring ~5.8 μm. Spermatids with flagella longer than 28 μm were all stained by the anti-TD02 antibody.

#### 3.2.5. Anti-Acetylated α-Tubulin Antibody

**Figure 6:**
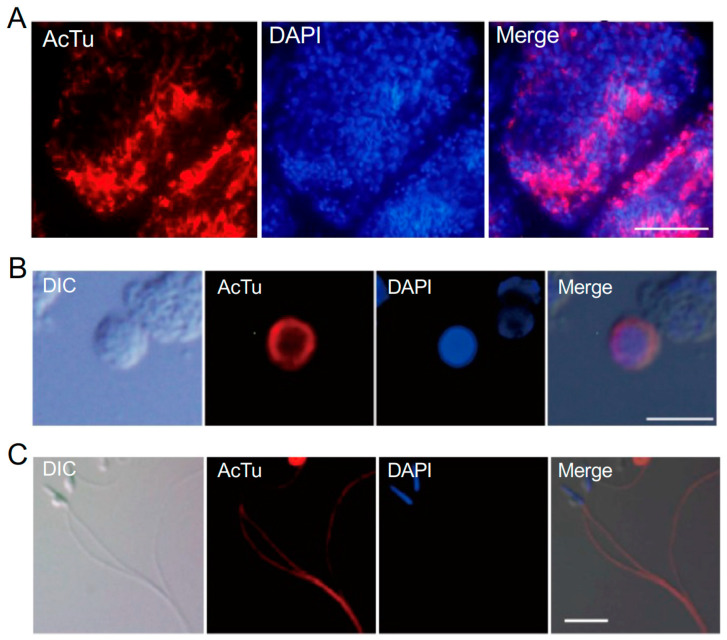
Immunofluorescence image of a frozen section stained with anti-acetylated α-tubulin antibody. (**A**) Immunofluorescence analysis of a testis section showed strong staining in the central area of the testicular follicle, similar to anti-TD02 staining. Scale bar, 20 μm. (**B**) The antibody recognizes the cytoplasm of non-flagellated spermatids (EI stage). Scale bar, 5 μm. (**C**) The flagella of mature sperm were strongly stained by the anti-acetylated α-tubulin antibody. Scale bar, 5 μm. Red, immunostaining with anti-acetylated α-tubulin antibody. Blue, DAPI staining.

Post-translational modifications of tubulins, such as acetylation, are critical for the formation and regulation of ciliary axonemes [28]. The acetylated α-tubulin antibody is commonly used to detect cilia and flagella [29]. The immunofluorescence analysis of testis sections showed strong staining in the central area of the testicular follicle, similar to anti-TD02 staining, but with a broader distribution, including non-flagellated spermatogenic cells (Figure 6A). In isolated cells, the antibody stained the cytoplasm of non-flagellated spermatids (Figure 6B). Approximately 75% of round-stage cells, with diameters ranging from 2.1 μm to 3.9 μm (RIII), were stained by the antibody. The flagella of spermatids and mature sperm were strongly stained (Figure 6C), consistent with previous findings in *Ciona* sperm [30,31].

### 3.3. Observation by Thin-Sectioned Electron Microscopy

**Figure 7:**
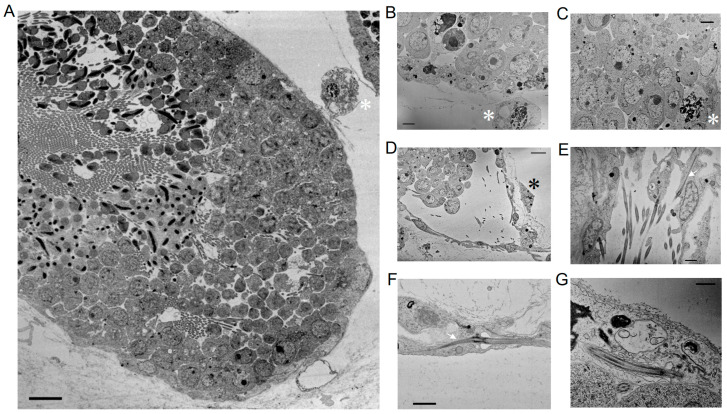
Transmission electron microscopy images of *Ciona* testis. (**A**) An image of a testicular follicle shows the non-cystic distribution of spermatogenic cells. A free cell with prominent heterochromatin in the nucleus (asterisk) is visible between testicular follicles. Scale bar, 5 μm. (**B**) A higher magnification of the free cell (asterisk). Scale bar, 2 μm. (**C**,**D**) Free cells (asterisks) are sometimes associated with the outer layer of a testicular follicle. Scale bars, 2 μm (**C**); 5 μm (**D**). (**E**,**F**) Long striated rootlets extend from the basal body of a flagellum in flat epithelial cells. Scale bars, 1 μm. (**G**) The axonemes exhibit a 9+2 structure with dynein arms, indicating motility. Scale bar, 500 nm. Arrows, striated rootlets.

Thin-sectioned electron microscopy confirmed the non-cystic distribution of spermatogenic cells in a testicular follicle (Figure 7A). We also confirmed the presence of free cells outside the follicle, which was recognized by the MIS H300 antibody (Figure 2). These cells are distinctly different from spermatogenic cells in that they have a nucleus with many spotted heterochromatin regions (Figure 7A,B). Some of these cells appeared to be tightly associated with the lamina of testicular follicles (Figure 7C,D). We also found the presence of ciliated cells in the epithelia of testicular follicles. The cilia were long and protruded from flat epithelial cells. A long striated rootlet extended from the basal body of a flagellum (Figure 7E,F). The cilia possessed motile 9+2 structured axonemes with dynein arms (Figure 7G).

## 4. Discussion

**Figure 8:**
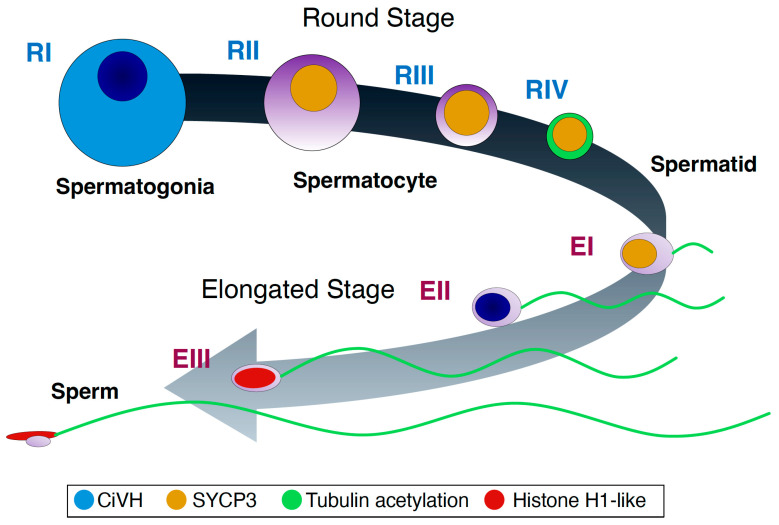
A schematic drawing of *Ciona* spermatogenesis based on the staining patters by several marker antibodies. Spermatogenic cells are categorized into four round stages (RI to RIV) and three elongated stages (EI to EIII). The reactivities against the antibodies used in this study and the localizations are indicated by colors. Stages at RI, RII/RIII and RIV/EI/EII/EIII are considered as spermatogonia, spermatocytes, and spermatids, respectively (see text).

Prior to this study, no characterization of spermatogenic cells in the ascidian *Ciona robusta* had been conducted at the molecular level. Here, we characterized these cells using antibodies against acetylated tubulin, Müllerian Inhibiting Substance (MIS), Vasa homolog, and the laboratory-made testis-specific proteins TD02 and TD09. Spermatogenic cells were classified into four round stages (RI to RIV) and three elongated stages (EI to EIII) (Figure 8). Based on previous morphological studies [17,18,20,22,23], cells in stages RI, RII/RIII, and RIV/EI/EII/EIII are identified as spermatogonia, spermatocytes, and spermatids, respectively. These antibodies clearly recognized structures specific to each stage, demonstrating that the method described here can be useful for further characterization of spermatogenic cells.

We further characterized the distribution of each spermatogenic cell type in the non-cystic testis of *Ciona*. Unlike cystic testes, where cells are encapsulated, the spermatogenic cells in *Ciona* are distributed as a mass within testicular follicles, with no clear boundaries between cell stages. Some cell populations are dispersed into adjacent areas. In vertebrates, the transition from lobule-type to tubule-type testes is thought to have occurred during evolution [3,32,33]. Lobule-type testes, seen in anamniotes such as fish and frogs, are characterized by cystic compartments that house specific stages of spermatogenic cells, each enclosed by Sertoli cells. In contrast, spermatogenesis in amniotes occurs within seminiferous tubules, where Sertoli cells are associated with spermatogenic cells throughout their development [3,33]. The lobule-type testis can be further divided into restricted and unrestricted lobules [32,33,34]. Three types of testes are observed in teleosts: tubule-type, restricted-lobule type, and unrestricted-lobule type. Lower teleosts, such as salmonids, exhibit tubular testis types, while higher teleosts have unrestricted lobular testes [32]. The arrangement of spermatogenic cells in the testicular follicles of *Ciona* resembles the tubular type found in lower fishes and vertebrates. Spermatogenesis in ascidians may provide key insights into the evolutionary emergence of cystic formation in testes.

We identified MIS-positive cells located outside the testicular follicles in *Ciona*. In lobule-type fish testes, the cystic formation of spermatogenic cells is thought to be supported by epithelial Sertoli-like cells [32]. In *Ciona*, these MIS-positive cells are scattered between the testicular follicles, likely corresponding to cells described in early gonad formation [19]. In sea urchins, another marine invertebrate, spermatogonia are located at the periphery of the gonad, and spermatogenesis proceeds from the epithelial basal wall toward the lumen in association with nutritive phagocytes [35,36]. It is possible that a primitive form of Sertoli cells, similar to nutritive phagocytes, emerged to support spermatogenesis before becoming fully associated with spermatogenic cells within cysts. The *Ciona* testicular follicle may represent a transitional stage from unorganized cell arrangements to a Sertoli-cell-assisted tubular testis structure. Further studies on the spatial organization and functional specialization of Sertoli cells in *Ciona* could shed light on an important event in the evolutionary transition from invertebrates to vertebrates within the chordate lineage.

We observed strong cytoplasmic staining with anti-acetylated tubulin antibody in round spermatid stages. Tubulin acetylation, which stabilizes microtubules, is typically localized in centrioles and ciliary axonemes. The cytoplasmic acetylation of tubulin was first reported in *Chlamydomonas* [29,37] and has since been observed during specific cell cycle stages in most cells, including axonal microtubules in neurons and certain microtubule structures in cancer cells [38]. In *Drosophila* spermatogenesis, which involves cytoplasmic ciliogenesis [39,40], acetylated tubulin is found in short elongating axonemes in the cytoplasm during the round spermatid stage [41], similar to our observations in *Ciona* testes. While the current study does not definitively show cytoplasmic ciliogenesis during *Ciona* spermatogenesis, it is possible that tubulin acetylation occurs prior to axoneme formation. Future studies should clarify whether cytoplasmic tubulin acetylation is associated with the pre-formation of doublet microtubules or is a prerequisite for their assembly from the basal body.

## 5. Conclusions

In this study, we classified spermatogenic cells in the *Ciona robusta* testis into seven distinct stages using immunostaining with several antibodies. The MIS H-300 antibody detected free cells between the testicular follicles, which are likely Sertoli-like cells that potentially support the differentiation of spermatogenic cells. Acetylated tubulins were identified not only in the flagella of elongating spermatids and mature sperm but also in round spermatids prior to flagellar elongation. This suggests that tubulin acetylation may play a role in the cytoplasmic formation of doublet microtubules or axonemes before the onset of flagellation. These findings provide new insights into the mechanisms of spermatogenesis and offer clues about the evolutionary transition of testicular structures in animals.

## Figures and Tables

**Table 1:** Classification of spermatogenic cell stages in *Ciona robusta*, based on both morphological and immunohistochemical characterizations.

		Spermatogenic Cells							Sperm
Spermatogenic Stage		Round Stage				Elongate Stage			
		RI	RII	RIII	RIV	EI	EII	EIII	
Cell Morphology	Cell Size (μm)	8.1~7.3 *	6.9~6.1	5.8~4.0	3.9~2.1	L: 3.44 ± 0.67 ** S: 2.68 ± 0.52	L: 3.17 ± 0.64 S: 2.52 ± 0.55	L: 3.19 ± 0.51 S: 1.78 ± 0.64	L: 3.7 S: 0.7
Cell Morphology	Nucleus Size (μm)	3.2 ± 0.30	3.11 ± 0.47	2.92 ± 0.39	2.28 ± 0.52	L: 2.22 ± 0.39 S: 1.81 ± 0.38	L: 2.25 ± 0.45 S: 1.81 ± 0.3	L: 2.65 ± 0.57 S: 1.31 ± 0.54	L: 3.4 S: 0.6
	Nuclear Shape	Spherical Elliptical	Elliptical	Spherical Elliptical	Spherical Elliptical	Spherical Elliptical	Spherical Elliptical	Spherical Elliptical Rod-shaped	Rod-shaped
	Flagellar Length (μm)	–	–	–	–	0~5.8	5.8~28.0	28.0~55	55
Immunohistochemical Staining	CiVH	◯ ***	×	×	×	×	×	×	×
	TD09	×	◯	◯	◯	△	×	×	×
	Ac-αTu	×	×	×	◯	◯	◯	◯	◯
	TD02	×	×	×	×	×	×	◯	◯
Corresponding Spermatogenic Stage		Spermatogonia	Spermatocyte		Spermatid				Sperm

* Numbers represent the size in μm. ** L or S indicates the length of long or short axis, respectively. *** Circle, cross or triangle shows that the cells are positively, negatively or partially stained by the antibody.

## Data Availability

Data are contained within the article.
